# Tea Tree Oil Terpinen-4-ol Protects Gut Barrier Integrity by Upregulation of Tight Junction Proteins *via* the ERK1/2-Signaling Pathway

**DOI:** 10.3389/fnut.2021.805612

**Published:** 2022-01-27

**Authors:** Yanhong Yong, Biao Fang, Yingxin Huang, Junyu Li, Tianyue Yu, Lianyun Wu, Canying Hu, Xiaoxi Liu, Zhichao Yu, Xingbin Ma, Ravi Gooneratne, Sidong Li, A. M. Abd El-Aty, Xianghong Ju

**Affiliations:** ^1^Shenzhen Institute of Guangdong Ocean University, Shenzhen, China; ^2^Department of Veterinary Medicine, College of Agricultural Sciences, Guangdong Ocean University, Zhanjiang, China; ^3^Department of Animal Science, College of Agricultural Sciences, Guangdong Ocean University, Zhanjiang, China; ^4^Department of Wine, Food and Molecular Biosciences, Faculty of Agriculture and Life Sciences, Lincoln University, Lincoln, New Zealand; ^5^College of Chemistry and Environment, Guangdong Ocean University, Zhanjiang, China; ^6^Department of Pharmacology, Faculty of Veterinary Medicine, Cairo University, Giza, Egypt; ^7^Department of Medical Pharmacology, Medical Faculty, Ataturk University, Erzurum, Turkey

**Keywords:** inflammatory bowel disease, terpinen 4-ol, ERK1/2-signaling pathway, tight junction (TJ) proteins, mouse model

## Abstract

Tea tree oil (TTO) exhibits a potent antioxidant, antibacterial, and anti-inflammatory activity and is commonly used in skincare products. However, it is not clear whether TTO can protect gut barrier damage in inflammatory bowel disease (IBD) patients. Herein, we report the impact of terpinen-4-ol (TER, the primary constituent of TTO), on lipopolysaccharide (LPS)-induced intestinal epithelial cell barrier function impairment in intestinal porcine epithelial cell lines (IPEC-J2) and dextran sulfate sodium (DSS)-induced IBD in mice. TER protected against LPS-induced damage in IPEC-J2 cells *in vitro* and attenuated DSS-induced colitis *in vivo*. Added TER promoted the tight junction (TJ) proteins expressing *in vitro* and *in vivo* and attenuated the LPS-induced upregulation of ERK phosphorylation in IPEC-J2 cells. However, when an inhibitor of ERK phosphorylation was added, TER did not promote the expression of TJ protein, denoting that the ERK signaling pathway mediates the upregulation of TJ proteins. Our data may propose the potential application of TER in treating IBD.

## Introduction

Inflammatory bowel disease (IBDs) is a chronic inflammatory disease of the gastrointestinal tract, which comprises Crohn's disease (CD), ulcerative colitis (UC), and indeterminate colitis (1). This disease affects all ages, and the clinical features primarily include fever, weight loss, diarrhea, and blood in stool (2). Although the exact causes of CD and UC are not well documented, previous studies have shown that genetic factors, immune system dysfunction, and environmental factors might play a crucial role in its occurrence (3). The lesions of IBD patients are mainly confined to the colorectal mucosa and submucosa. They are characterized by mucosal barrier damage and impaired tight junction (TJ) functions, resulting in a loss in gut barrier integrity (4).

Tight junctions between epithelial cells play a role in maintaining the permeability and integrity of the intestinal mucosal barrier. Occludin, claudins, and zonula occludens-1 (ZO-1) are the essential adhesion proteins responsible for the efficient functioning of TJs in the intestinal lining (5). The intestinal mucosal barrier is damaged in IBD patients, and the expression of the TJ proteins ZO-1, claudin, and occludin was decreased in the intestine (6). Otherwise, the upregulation of both ZO-1 and occludin expression significantly improves the integrity, reduces the intestinal mucosal barrier permeability, and prevents the infiltration of harmful substances in IBD patients (7, 8).

There are no drugs to cure IBD, and, at best, they serve to minimize the disease process (9). Drugs commonly used for IBD treatment, include corticosteroids, immunosuppressants, and immunomodulators, which may induce adverse effects on long-term usage (10). Therefore, it has been proposed that IBD patients be treated to restore the mucosal barrier function and thereby relieve the clinical symptoms and cure IBD (11). Given that TJ protein expression levels are closely related to the mucosal barrier function, we speculated that developing a “natural” drug that can normalize TJ function may be an approach for treating IBD.

Tea tree oil (TTO) is extracted from *Melaleuca alternifolia*. Because of its broad-spectrum antimicrobial (bacteria, fungi, and viruses), anti-cancer, anti-tussive, and antioxidant properties (12). TTO is widely used to treat acne, athlete's foot, and contact dermatitis. TTO contains over 100 components, including terpineol, zinc, and diheptoxy-sulfanylidene-sulfido-λ 5–phosphane (13), the most crucial being terpinen-4-ol (TER), which accounts for >30% of all TTO components. Currently, TER is widely used in the food industry. For instance, as an antistaling agent, TER can alter the microbial biofilm formation in foods, thereby preventing food deterioration and reducing the incidence of certain foodborne illnesses (14). TER has inhibitory activity against certain aerobic heterotrophic bacteria in food (15). It has also been used as a spice to reduce breast cancer incidence (16). Mechanistically, TER can downregulate the secretion of the inflammatory signaling molecule, NO, which is induced by lipopolysaccharide (LPS) and dioctyl sodium sulfosuccinate (DSS) (17, 18). In addition, TER plays a role in regulating the mitogen-activated protein kinase (MAPK)-signaling pathway (19), which is intimately involved in the expression of TJ proteins (20). Hence, we hypothesized that TER would be a promising candidate for IBD therapy. In this study, we examined the effects and mechanisms of TER in protection against LPS/DSS-induced inflammation, both *in vitro* and *in vivo*.

## Materials and Methods

### Fourier-Transform Infrared Spectroscopy (FTIR) Analysis

TER was a gift from Professor Sidong Li's laboratory. His group isolated TER from TTO, the essential oil extracted from *Melaleuca alternifolia*. The protocol of FTIR analysis was conducted as described by Linshi and modifications (21). Briefly, samples were dissolved in heavy water (D_2_O) and detected with a Bruker Tensor 27 infrared spectrometer (Shanghai, China) with a resolution of 4.0 cm^−1^ and a scan range of 400–4,000 cm^−1^. An average of 64 scans was required to obtain information about the structure.

### Cell Culture

IPEC-J2 cells were generously donated by Dr. Bruce Schultz of Kansas State University. The cells were seeded in a T25 flask (Corning Inc., Corning, NY, USA) and cultured in an incubator at 37 °C and 5% CO_2_. The cells were grown in DMEM/F12 (Sigma-Aldrich, St. Louis, MO, USA) added with 100 U/mL penicillin, 100 μg/mL streptomycin, and 10% fetal bovine serum. After the cells were grown to sub-confluence in 24-well plates, the culture medium was removed, and the cells were washed twice with phosphate-buffered saline (PBS). Subsequently, the cells were exposed to LPS with or without TER.

### Cell Viability Assay

A Cell Counting Kit-8 (CCK8, MedChemExpress, Shanghai, China) was used to detect the viability of cells as described by Linshi and modifications (21). Briefly, the IPEC-J2 cells were scattered into 96-well plates and cultured for 24 h, then treated with TER (0, 0.001, 0.002, 0.004, 0.006, 0.008, 0.01, 0.016, and 0.02%) for another 24 h, then incubated with 10 μL of CCK8 solution. Next, the absorbance at 450 nm was detected by the plate reader.

### Determination of Epithelial Cell Integrity

The protocol of epithelial cell integrity detection was according to the methods reported by Linshi et al. (21). Briefly, cells were scattered at a 1 × 10^5^ cells/mL density into Trans-well-COL (Corning). The cells were cultured in a 37 °C, 5% CO_2_ incubator for 13–15 days until they reached a complete polarization, and the culture media was refreshed per 2 days. After the fused monolayer epithelial cells were formed, serial concentrations (as described above) of TER were added and treated for 24 h. Next, 10 μg/mL LPS was added to Trans-well plates' upper compartment (22). The Millicell ERS-2 Voltohmmeter (Millipore, Billerica, MA, USA) were used to measure Trans-epithelial electrical resistance (TEER) of monolayer epithelia at 0, 12, and 24 h. 4-kDa fluorescein isothiocyanate-labeled dextran (FITC-dextran, Sigma-Aldrich). FITC-dextran was dissolved in DMEM/F12 and added to the upper compartment with a 2.2 mg/mL concentration. Two hours later, the lower compartment fluorescence intensity was detected by fluorometry (Tecan Group, Switzerland; excitation, 490 nm; emission, 520 nm).

### qRT-PCR

The total RNA of cells and tissue were extracted by the standard method using TRIzol^®^ Reagent (Takara, Dalian, China). The quality of extracted RNA was analyzed by the ratio of A260/A280 using spectrometry. Then, the RNA was reverse transcribed to cDNA using the PrimeScript RT reagent kit (Takara, Dalian, China). Next, the SYBR^®^ Premix Ex Taq™ II (Takara, Dalian, China) were used to perform qRT-PCR. The gene expression level was analyzed by the ΔΔCT method (23). The primers used in the reaction are shown in Table 1.

**Table 1:** Primer sequence.

**Primer**	**Sequence (5^**′**^-3^**′**^)**
claudin-1 forward	GTGGATGTCCTGCGTGTC
claudin-1 reverse	GTGTTGGGTAAGATGT TGTTTT
claudin-4 forward	TTCATCGGCAGCAACAT
claudin-4 reverse	AGGACACGGGCACCAT
Occludin forward	ATCAACAAAGGCAA
Occludin reverse	CTCCGTAATGACCAGA
ZO-1 forward	GCCTCCTGAGTTTGATAGTG
ZO-1 reverse	TCGGCAGACCTTGAAATAGA
β-actin forward	TGCGGGACATCAAGGAGAAG
β-actin reverse	AGTTGAAGGTAGT TTCGTGG

### Western Blotting

The western blotting was performed as Linshi reported methods and modification. RIPA Lysis Buffer (Beyotime Biotechnology, Shanghai, China) and extraction kits (ThermoFisher Scientific, Shanghai, China) were used to isolate the total proteins, a nuclear protein, and cytoplasmic proteins, respectively. The protein concentration was analyzed using a BCA reagent (CWBIO, Beijing, China) followed by SDS-PAGE and electrotransfer of separated protein lysates to nitrocellulose membranes (Millipore). After 1 h of blocking, the membranes were incubated with a primary antibody at 4°C for overnight, then incubated with the secondary antibody for 2 h at room temperature. Positive bands were measured by enhanced chemo-luminescence (Tanon, Shanghai, China). Gel-Pro Analyzer software version 4.0 (Media Cybernetics, Silver Spring, MD, USA) was used to analyse the densitometry. Antibodies against ZO-1 (ab96587), occludin (ab167161), claudin-1 (ab129119), and claudin-4 (ab15104) were obtained from Abcam (Cambridge, MA, USA). The antibody against ERK (4695S), P-ERK (4370S), and β-actin (4970S) were secured from CST (CST, MA, USA).

### Enzyme-Linked Immunosorbent Assay

The cell culture supernatant was stored at −20 °C before analysis. According to the manufacturer's instructions, the IL-6 concentration and TNF-α concentration were detected by ELISA Kit (IL-6, P6000B; TNF-α, PTA00; R & D Systems, Inc. Minneapolis, MN, USA). The plates were read by a microplate reader (BioTek Instruments, INC, USA) at 405 nm wavelength.

### Animal Experiments

**Figure 1:**
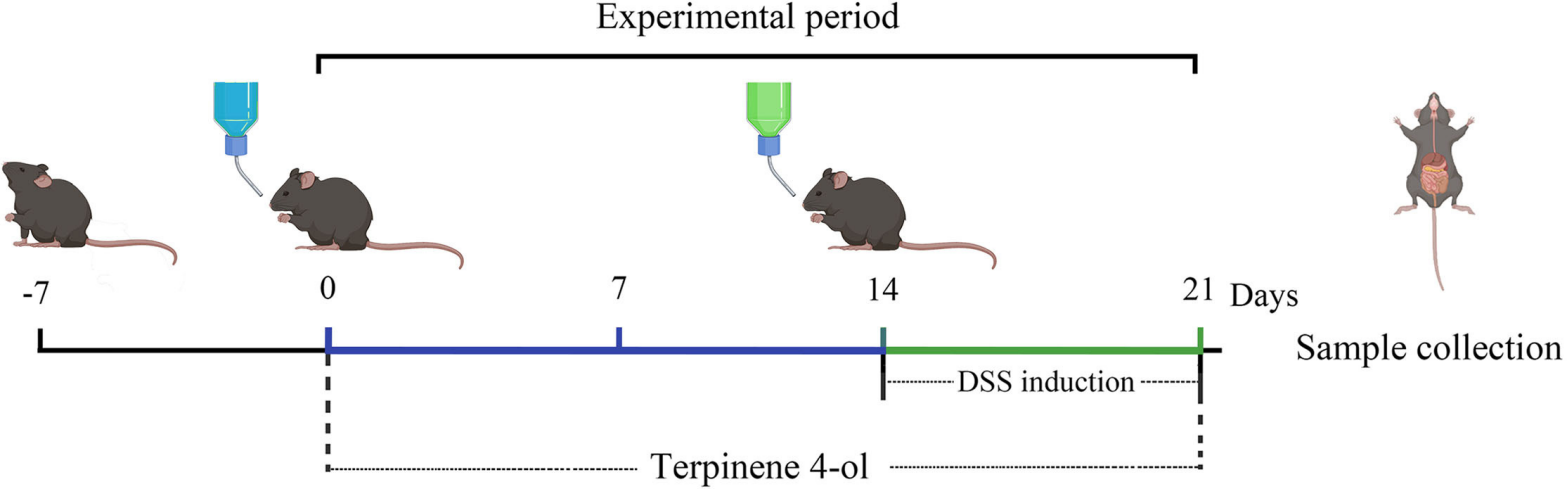
Experimental design demonstrating the effect of terpinen-4-ol (TER) on ameliorating colitis.

The six-week-old male mice (C57BL6/J) were reared in the Animal Housing Unit and controlled environmental temperature around 24 ± 1°C with a 12-h light/12-h dark cycle. The experimental protocols used were as follows (a brief protocol is shown in Figure 1). The mice were randomly allocated into one of six groups (five animals per group), namely the control, PBS (PBS, i.p.), DSS, DSS + TER-L (5 mg·kg^−1·^day^−1^, i.p.), DSS + TER-M (10 mg·kg^−1·^day^−1^, i.p.), and DSS + TER-H (20 mg·kg^−1·^day^−1^, i.p.). After TER pre-treatment, DSS (2%, *v/w*, MW 36,000–50,000, MP Biomedicals, Aurora, OH) was added to drinking water from days 15 to 21. All experimental protocols were approved by the Animal Ethics Committee of Guangdong Ocean University, China (Clearance No. 2018-0008) and performed according to the European Community Ethical Guidelines (Directive 2010/63/EU).

### Assessment of Colitis

The method for colitis assessment was followed by Linshi's reported and minimal modification (21). The animal's body weights (BWs) were recorded daily. All animals were euthanized on day 21 with CO_2_, and the blood, colon, and colon contents were collected. Two 5-mm sections of each colon were obtained and fixed in 4% paraformaldehyde and Carnoy's fixative (dry methanol: chloroform: glacial acetic acid at a volume ratio of 60:30:10). The rest of the colon was rinsed with saline and stored at −80°C pending analysis. After fixation, colon samples were dehydrated and embedded in paraffin wax. 5 μm slices were prepared using a microtome (Thermo Fisher) and stained with haematoxylin/eosin (H&E) and periodic acid–Schiff (PAS). The stained slices were covered with coverslips using neutral balsam as an adhesive. The thickness of the muscle layer, the height of the villus, and the number of goblet cells was calculated by Image-Pro Plus software, version 6.0 (Media Cybernetics, Silver Spring, MD, USA).

### Statistical Analyses

GraphPad Prism^®^ 5.0 (GraphPad Software, La Jolla, CA, USA) was used for all statistical analyses and graphs. Error bars refer to the standard error of the mean (SEM). The means ± SEM presented are from at least three experiments. The two means were compared using Student's *t*-test.

## Results

### Chemical Profile of TER

**Figure 2:**
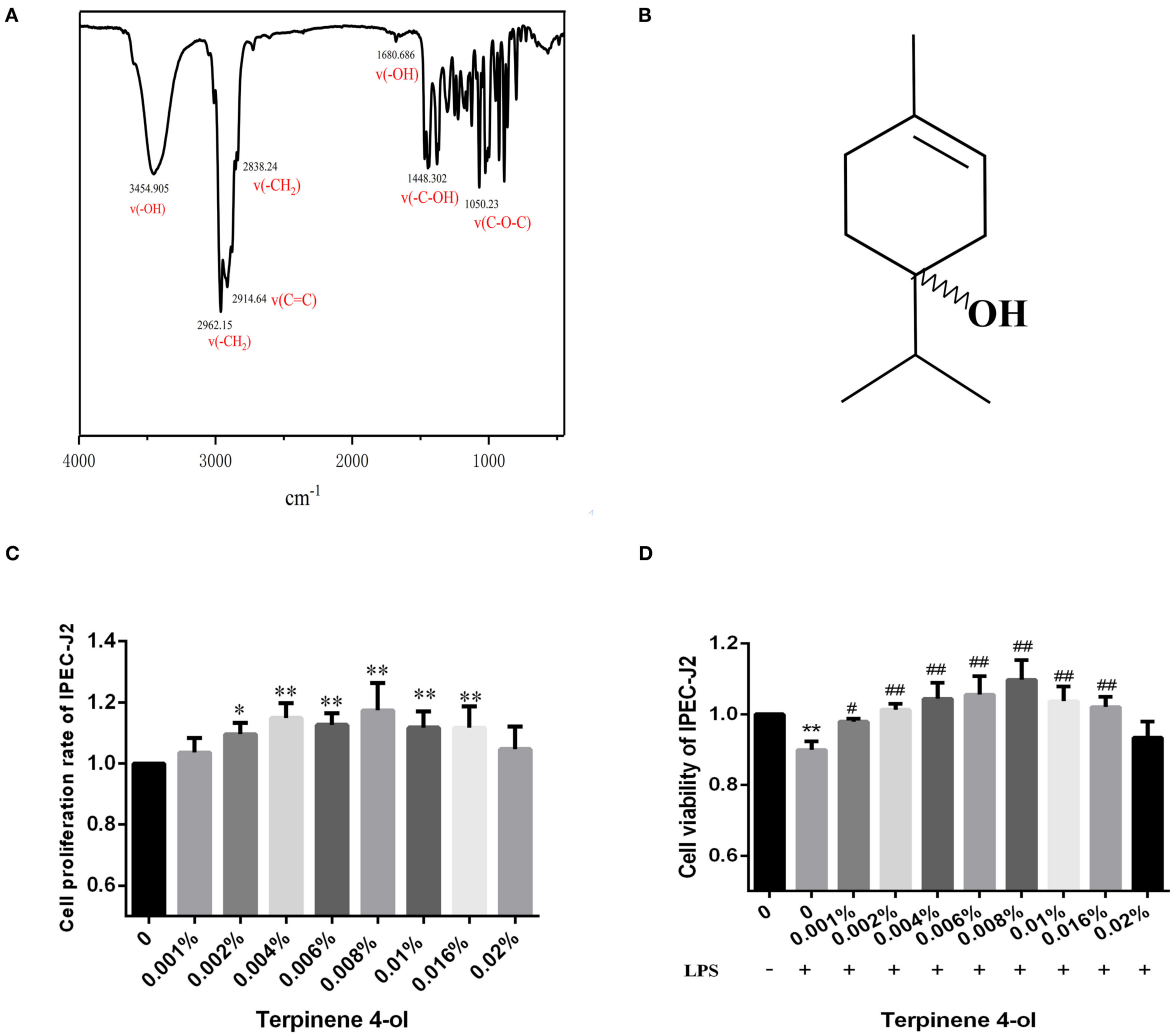
The characterization of TER. **(A)** The infrared spectrum of TER. **(B)** The chemical structure of TER intestinal epithelial cells pretreated with different TER concentrations for 24 h followed by treatment with LPS (10 μg/mL) for 24 h. The effects of TER on **(B,C)** cytotoxicity and **(D)** cell viability were observed in IPEC-J2 cells exposed to LPS. The data are expressed as the mean ± standard error of three independent experiments. * *P* < 0.05, ** *P* < 0.01, compared with the blank control group. # *P* < 0.05, ## *P* < 0.01, compared with the blank LPS group.

As assessed by FTIR spectroscopy, the structure characteristics and molecular mass of TER are shown in Figures 2A,B. The IR spectra revealed detection at 3,454 cm^−1^ (-OH stretching); 2,962 cm^−1^, 2,838 cm^−1^ (-CH_2_ stretching); 2,914 cm^−1^ (C=C stretching); 1,448 cm^−1^ (-C-OH); 3,400 cm^−1^ (-OH stretching vibration); 1,680 cm^−1^ (-OH bending); and 1,050 cm^−1^ (C-O-C stretching). The spectral results were similar to the TER structure reported in previous studies (24, 25).

### Cell Viability After Exposed to TER

Cells were treated with different TER concentrations for 24 h to determine the highest non-toxic TER concentration. Figure 2C shows that TER did not significantly inhibit IPEC-J2 cell proliferation. At concentrations between 0.002% and 0.016%, the viability rate of IPEC-J2 significantly increased (*P* < 0.05). The viability rate of IPEC-J2 cells significantly decreased following LPS exposure (Figure 2D). However, upon TER pretreatment, the cell viability rate increased significantly (*P* < 0.05). The most effective TER concentrations ranged between 0.001% and 0.016%.

### Effect of TER on the Intestinal Epithelial Cell Monolayer Integrity Exposed to LPS

**Figure 3:**
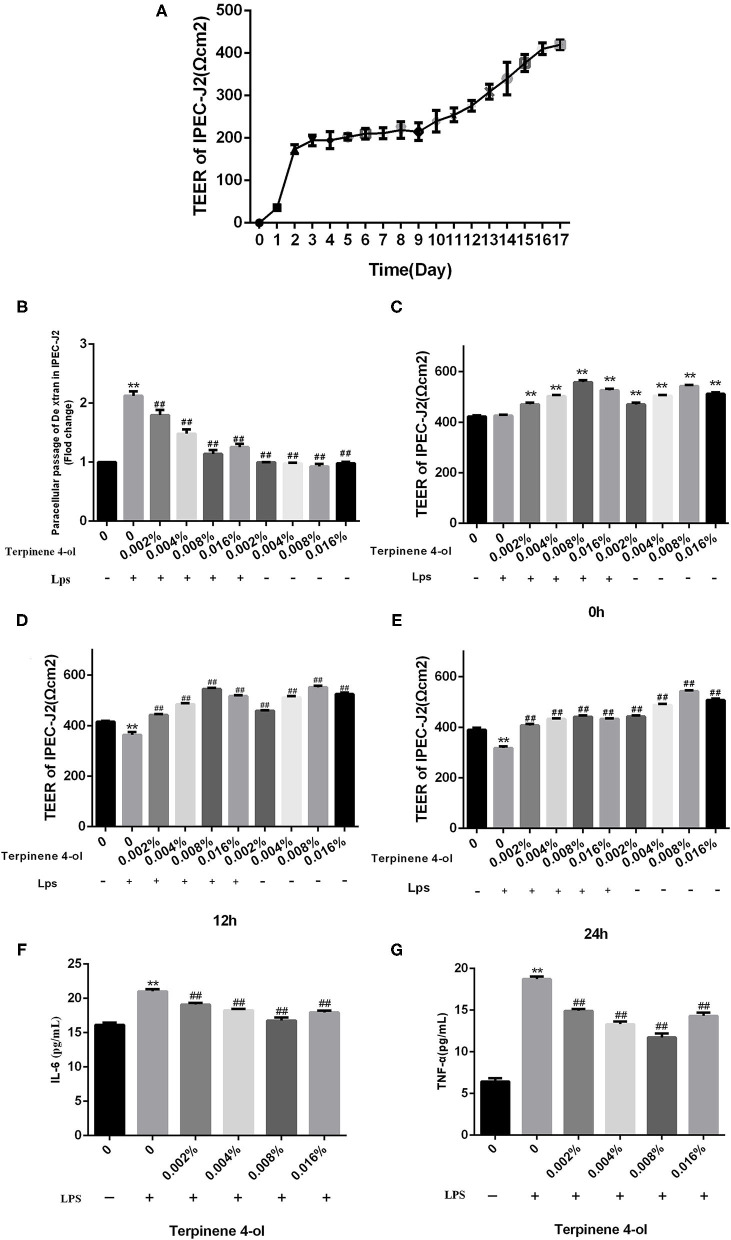
Effects of TER on LPS-induced intestinal epithelial cell permeability and inflammatory cytokines. The protective effects of TER on fused monolayers of intestinal epithelial cells were examined by measuring the TEER values of fused intestinal epithelial monolayers at different time points and FITC-dextran permeability. (A) TEER values. Cells were treated with LPS on day 17 after differentiation, and TEER values were detected at different times **(B–D)**. Cell permeability was measured by determining FITC-dextran permeability after 24 h of LPS exposure in TER-treated cells **(E)**. The IL-6 **(F)** and TNF-α **(G)** levels in the culture supernatants of cells pretreated with TER for 24 h and then exposed to LPS for 3 h were measured by ELISA. ** and ## display similar means with above mentioned.

The effects of TER on intestinal epithelial cell permeability were determined by gauging TEER values and concentration of FITC-dextran after being exposed to LPS. The TEER values of IPEC-J2 epithelial cells fused monolayers were stable at 16 d after seeding, indicating that the single-layered epithelium fusion was successfully constructed (Figure 3A). The TEER values of the epithelial monolayer significantly increased after pre-treating with different TER concentrations for 24 h. When fused IPEC-J2 epithelial cell monolayers were exposed to LPS (10 μg/mL), the TEER values significantly decreased (*P* < 0.05) and the permeability to FITC-dextran was remarkably increased (*P* < 0.01). Adding different concentrations of TER significantly (*P* < 0.01) reversed the LPS-dependent decrease in TEER values and increased FITC-dextran permeability in IPEC-J2 monolayers (Figures 3B–E).

### Effect of TER on IL-6 and TNF-α Expression in IPEC-J2 Cells Exposed to LPS

The cells were centrifuged, and the supernatant was collected to detect the expression of IL-6 and TNF-α. As shown in Figures 3F,G, the IL-6 and TNF-α levels, all of which were significantly increased (P < 0.01) in the supernatants of IPEC-J2 cells challenged by LPS, but were significantly decreased (*P* < 0.01) when pre-treated with TER.

### Effect of TER on TJ Proteins Expressing in IPEC-J2 Cells Exposed to LPS

**Figure 4:**
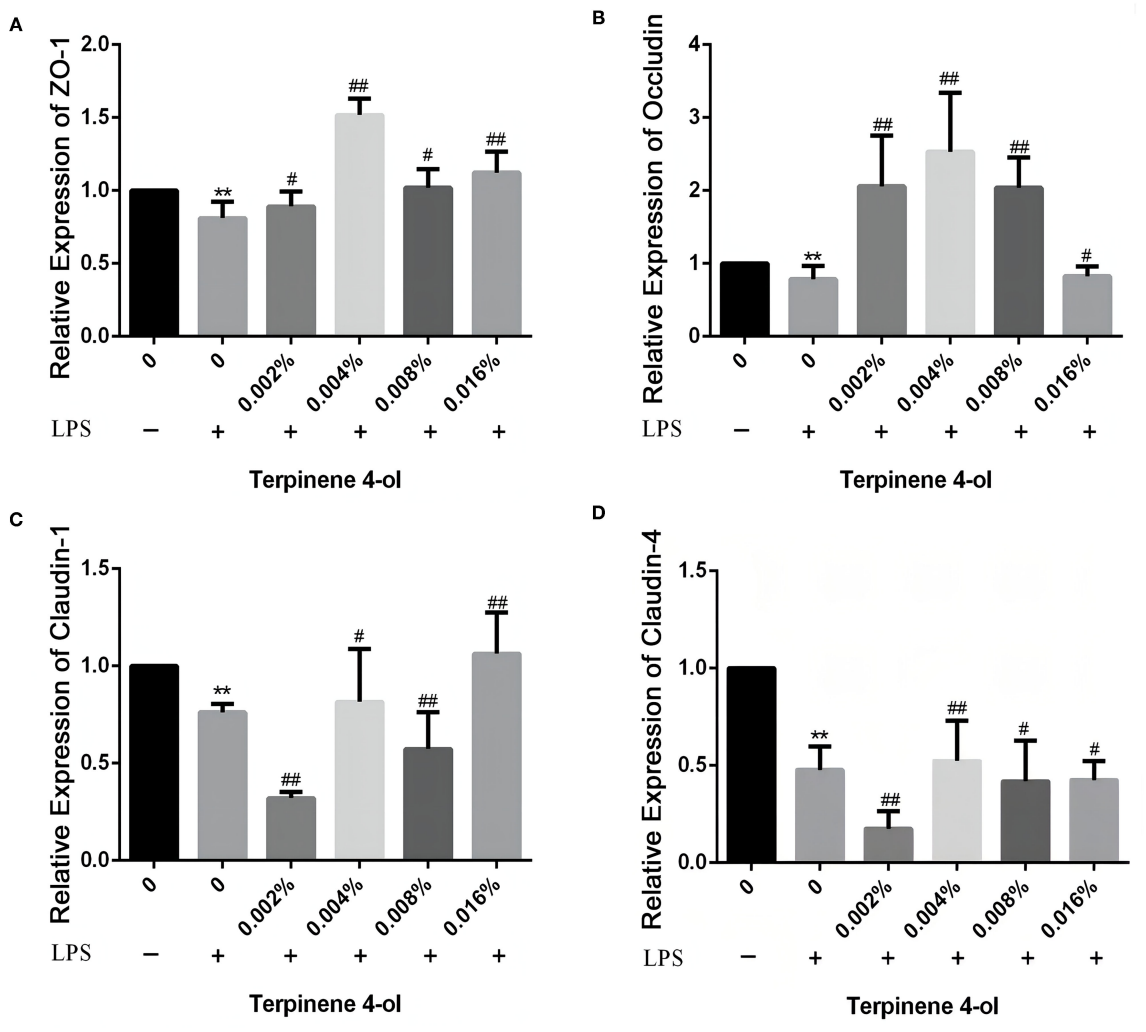
Effect of TER on tight junction proteins in IPEC-J2 cells. The expression of ZO-1 **(A)**, occluding **(B)**, claudin-1 **(C)**, and claudin-4 **(D)** genes were analyzed by qRT-PCR. ** and ## display similar means with above mentioned.

**Figure 5:**
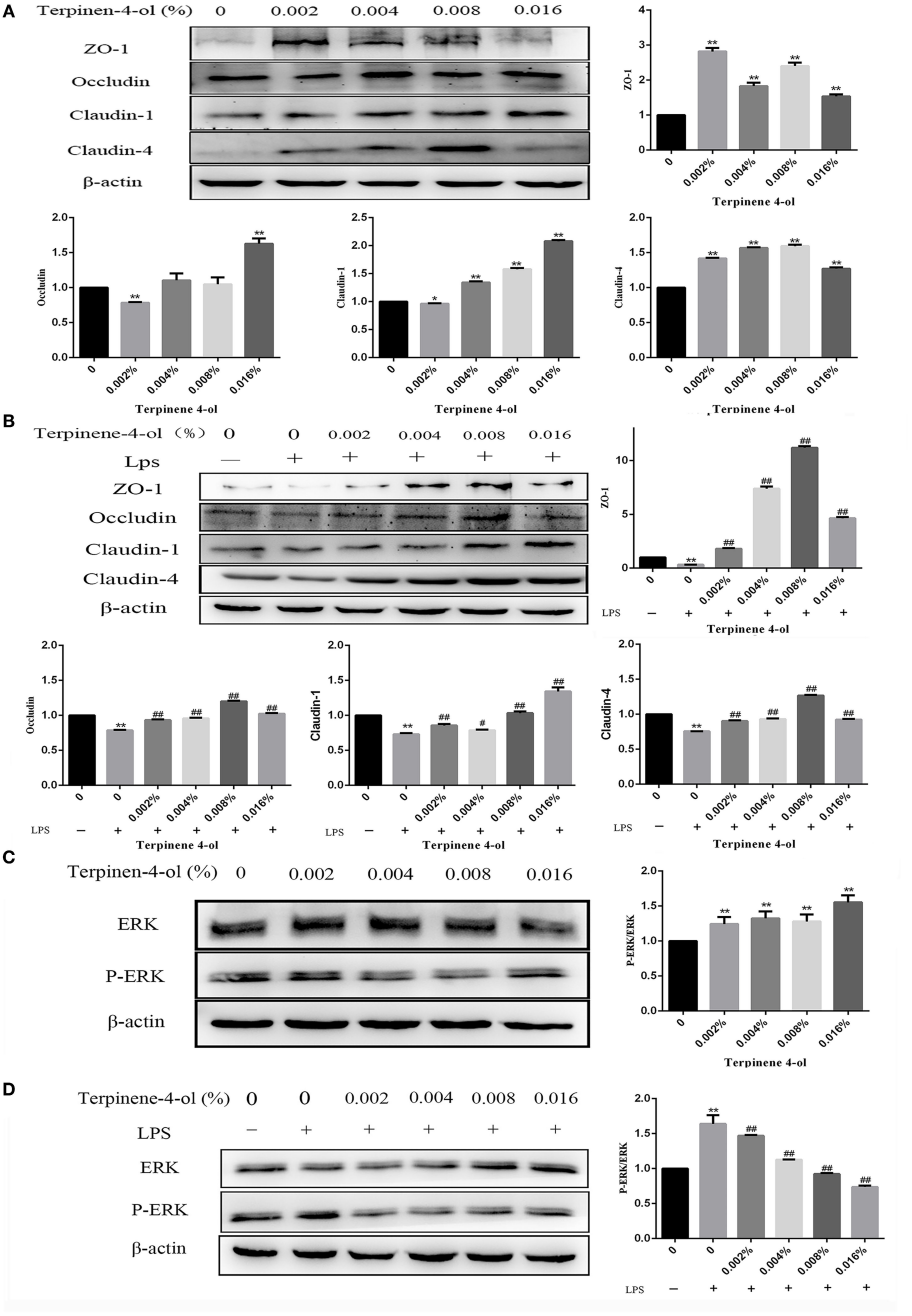
Effect of TER on tight junction (TJ) proteins and phosphorylated ERK expression in IPEC-J2 cells. **(A)** The TJ proteins expression were detected in cells exposed to a serial concentration of TER. **(B)** The TJ proteins' expression in cells incubated with TER for 24 h and then exposed to LPS. **(C)** ERK and phosphorylated ERK expression in cells incubated with TER for 24 h. **(D)** ERK and phosphorylated ERK expression in cells incubated with TER for 24 h and then exposed to LPS. ** and ## display similar means with above mentioned.

Following 24 h pretreatment with TER, the TJ protein expression in IPEC-J2 cells were measured by qPCR and western blotting. TER significantly upregulated the expression of tight junction proteins, such as ZO-1, occludin, claudin-1, and claudin-4. Interestingly, when cells were exposed to LPS for 3 h, the above-mentioned proteins level was significantly decreased (*P* < 0.05). However, TER pretreatment significantly inhibited LPS-induced decreased TJ proteins at both mRNA (Figures 4A–D) and protein (Figures 5A,B) levels.

### Effects of TER on ERK Protein and Its Phosphorylation in IPEC-J2 Cells

Following 24-h pretreatment with TER, ERK and phosphorylated ERK in cells were measured by immunoblotting. TER treatment was significantly downregulated (*P* < 0.05) the expression of phosphorylated ERK in IPEC-J2 cells (Figure 5C). Following exposure to LPS for 3 h, the phosphorylated ERK levels were significantly increased (*P* < 0.05). However, pre-treatment of the cells with TER for 24 h and subsequent exposure to LPS resulted in a phosphorylated ERK decrease (Figure 5D, *P* < 0.05).

### Effects of Blocking ERK Phosphorylation on TJ Protein Expression

**Figure 6:**
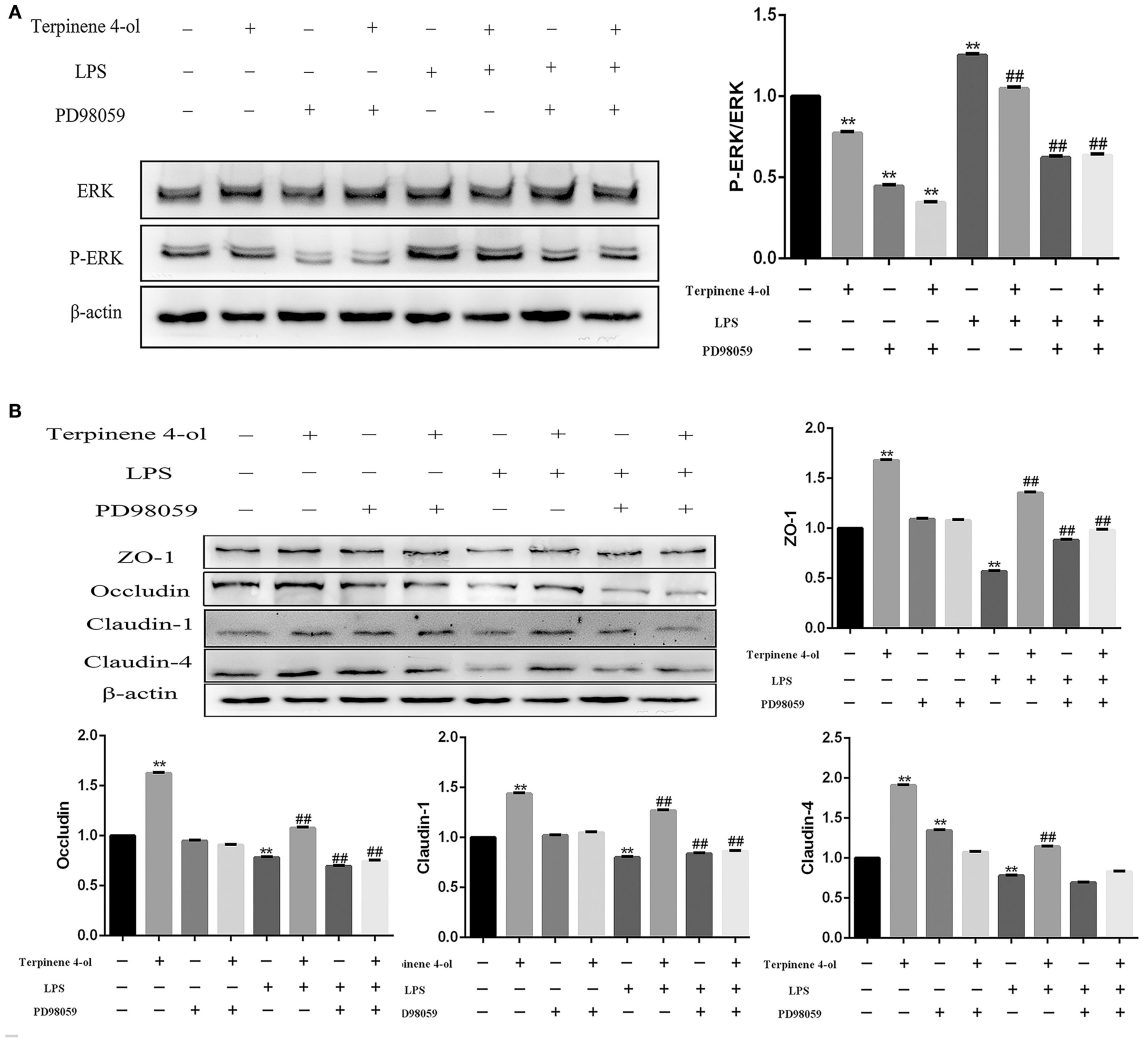
Effect of ERK phosphorylation inhibition on tight junction (TJ) protein expression. **(A)** The expression of phosphorylated ERK and **(B)** TJ proteins were detected in cells pretreated with the ERK inhibitor PD98059 (50 μM) for 1 h before treatment with TER or LPS. ** and ## display similar means with above mentioned.

To examine the role of ERK in regulating TJ proteins expression, cells were incubated with an ERK inhibitor (PD98059, 50 nM) for 1 h before treatment with TER (0.008%) or LPS (10 μg/mL). The level of phosphorylated ERK was significantly decreased (*P* < 0.01) after adding the inhibitor (Figure 6A), suggesting that the ERK pathway was successfully blocked. Although the expression of TJ proteins was upregulated significantly (*P* < 0.01) in cells pre-treated with TER alone, but the ERK inhibitor significantly attenuated (*P* < 0.01) the upregulation of TJ proteins induced by TER. In TER-pretreated cells that were later exposed to LPS, the inhibitor significantly decreased (*P* < 0.01) the expression of TJ proteins (Figure 6B).

### Effects of TER on DSS-Induced Colonic Inflammation in Mice

**Figure 7:**
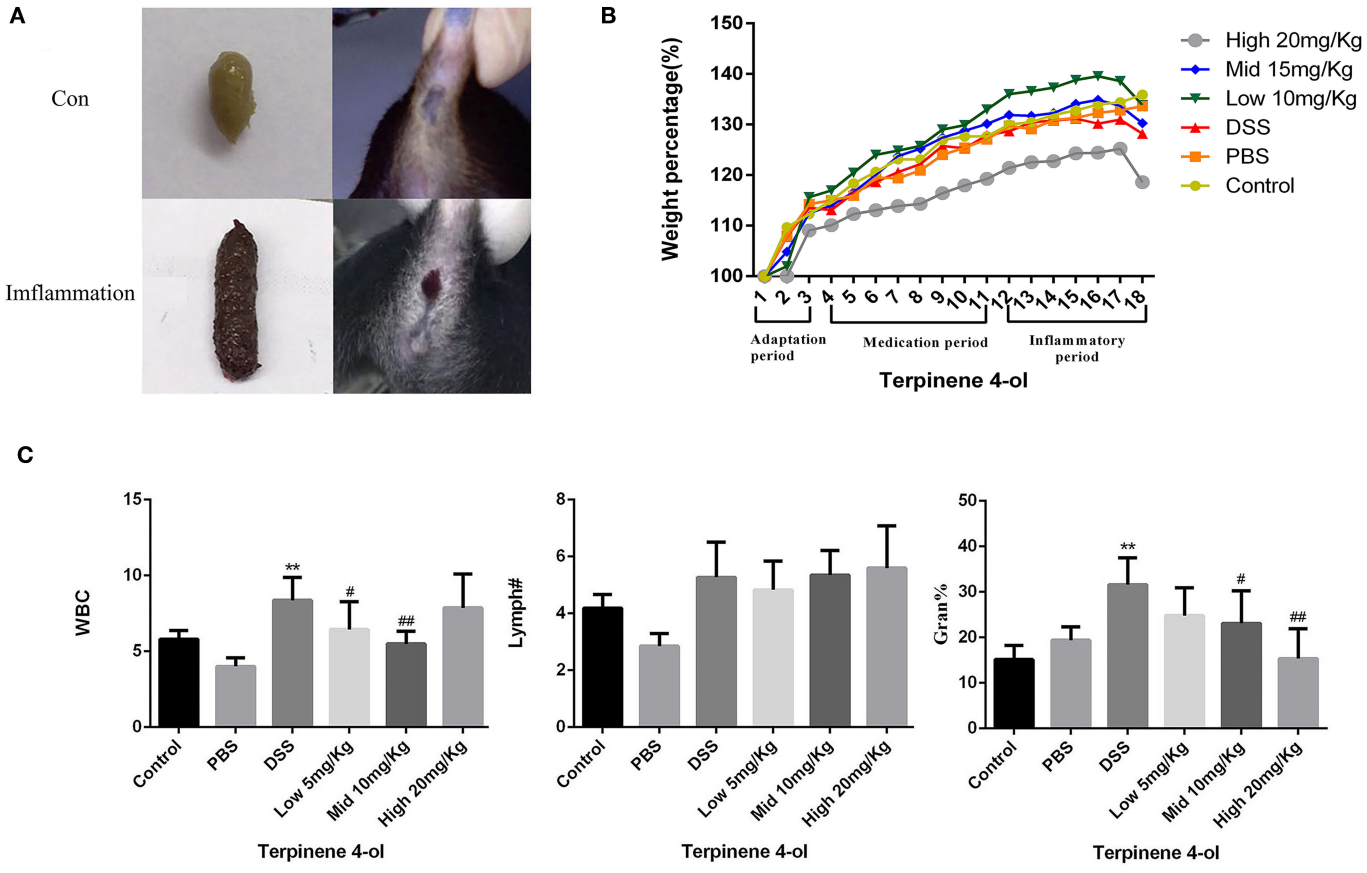
Effect of TER on colonic inflammation in mice with DSS-induced colitis. Colonic inflammatory responses after drinking DSS solution. **(A)** Severe bloody stools, **(B)** relative BW changes, **(C)** and the percentages of white blood cells, lymphocytes, and neutrophils in mice treated with TER. ** and ## display similar means with above mentioned.

The mice given DSS-containing water showed reduced food intake, lethargy, dry hair, slow responses to stimulation and impotence. Severe bloody stools and/or anal bleeding was also noticed (Figure 7A). The BWs of mice in the control and the PBS-treated groups increased steadily, whereas it decreased in those administered DSS alone on day 3 post-treatment. Lower BW losses were observed in mice exposed to both low- and moderate- levels of TER than in the DSS group in contrast to higher BW losses in mice exposed to a high TER level (Figure 7B). Routine blood test results showed that the proportions of leukocytes, lymphocytes, and neutrophils increased in the DSS-treated mouse. However, TER (low and moderate doses) administration attenuated the increase of inflammatory response induced by DSS in mice (Figure 7C).

### TER Attenuated Mice Colitis Induced by DSS

**Figure 8:**
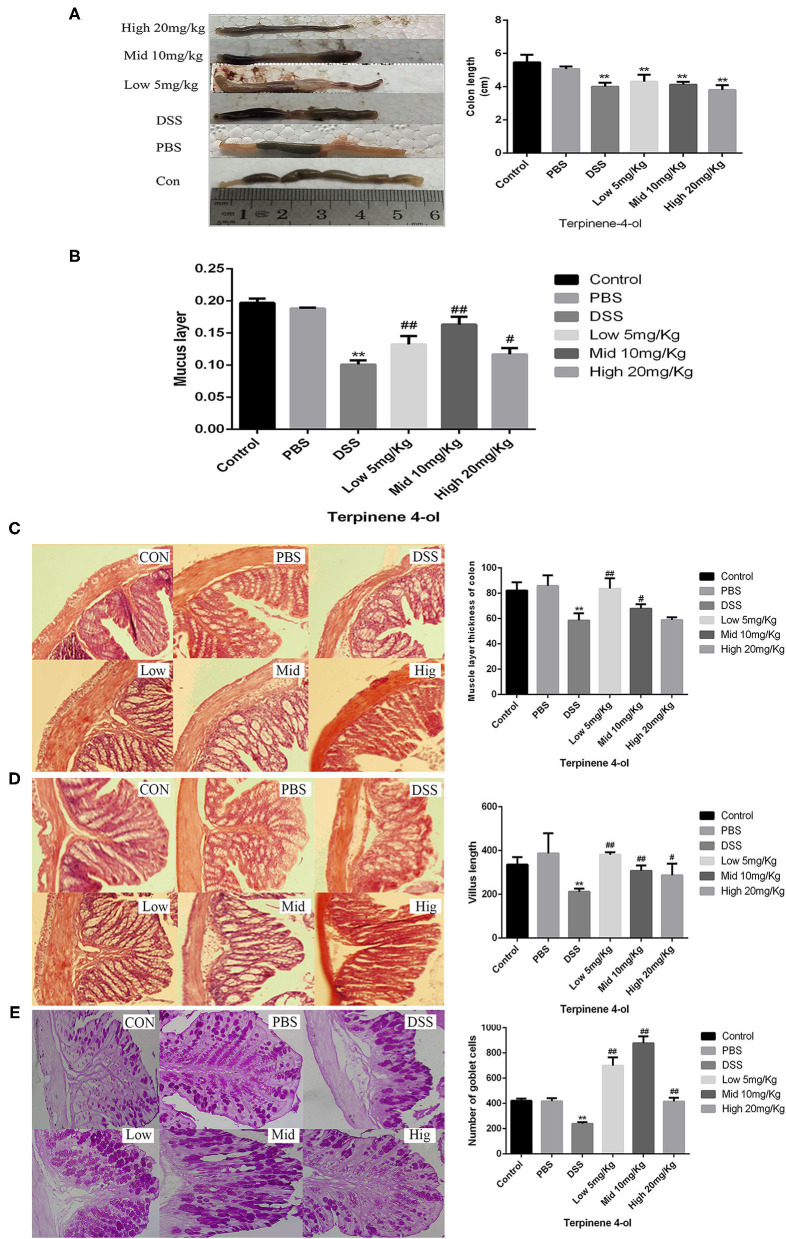
Effect of terpinen-4-ol (TER) treatment on colonic histopathology and the intestinal mucosal layer thickness in dextran sulfate sodium (DSS)-induced colitis. Effect of TER treatment on the colon length **(A)**, mucus layer thickness **(B)**, muscle layer thickness **(C)**, villus length **(D)**, and the number of goblet cells **(E)** in mice with DSS-induced colitis. ** and ## display similar means with above mentioned.

The colon structure in control mice was normal under the microscope. However, in DSS-treated mice, significant inflammatory responses, such as the short of colon length (Figure 8A), the disappear of intestinal mucous layer (Figure 8B), the thickness of the muscle layer of the colon (Figure 8C), the short of intestinal villus (Figure 8D), and marked decrease of the number of goblet cell (Figure 8E) have appeared. Interestedly, both lower concentration and middle concentration of TER added protected the goblet cells and the mucosal integrity in DSS-induced colitis mice.

### Effect of TER on TJ Proteins and ERK-Pathway Protein Expression *in vivo*

**Figure 9:**
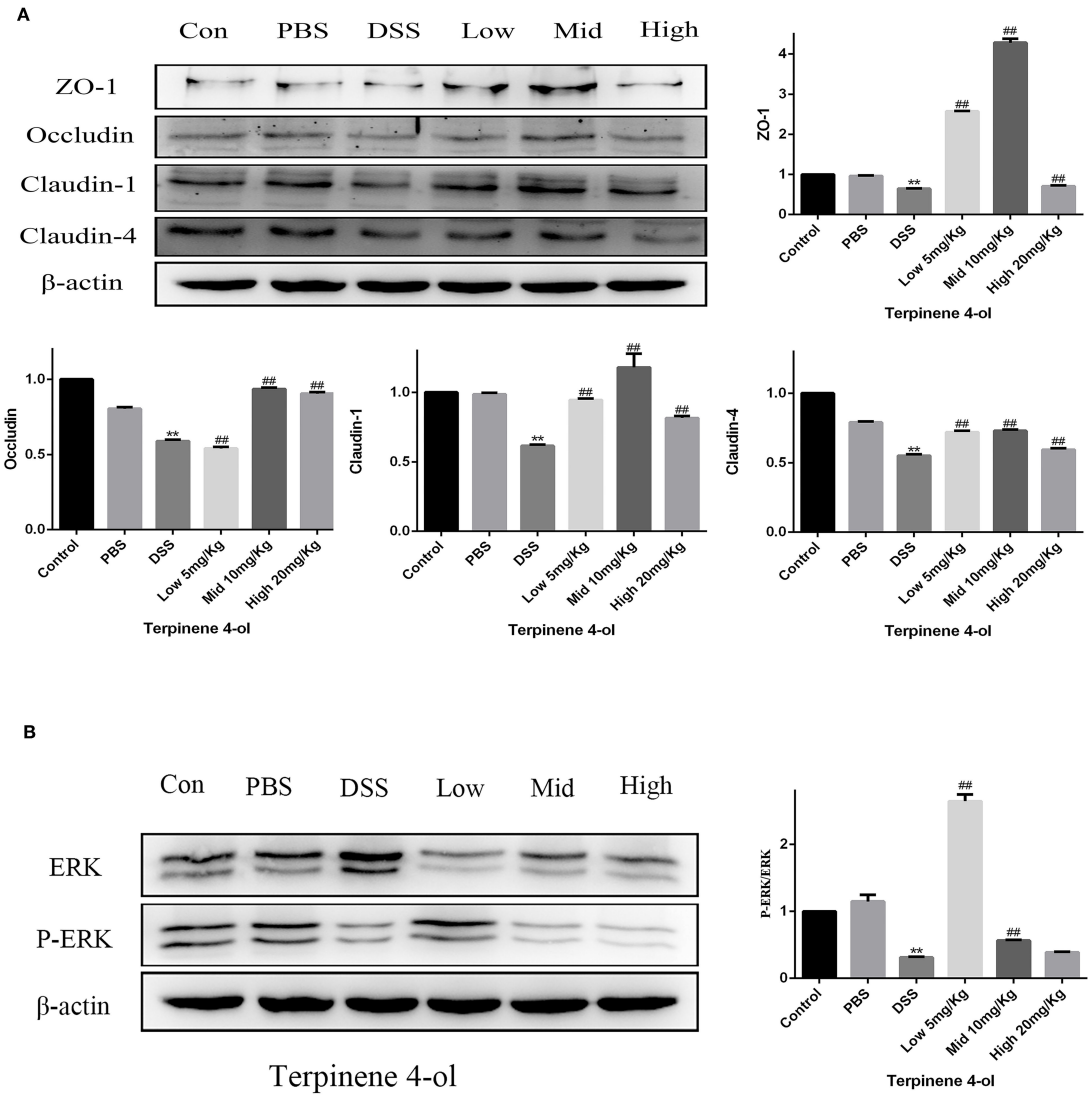
Effect of terpinen-4-ol (TER) on the tight junction (TJ) and ERK-pathway protein expression in mice. **(A)** Expression of colon TJ proteins and **(B)** ERK-signaling proteins in mice after drinking DSS solution. ** and ## display similar means with above mentioned.

Compared with the control and PBS groups, TJ protein expression levels were markedly reduced (*P* < 0.05) in the DSS treated group but increased in TER pre-treated groups (Figure 9A, *P* < 0.05). Meanwhile, the expression of phosphorylated ERK was remarkably decreased in the DSS treated group compared with control but increased in mice with a lower dose of TER administration (Figure 9B, *P* < 0.05), suggesting that TER upregulated the expression of tight junction proteins was related to phosphorylated ERK.

## Discussion

**Figure 10:**
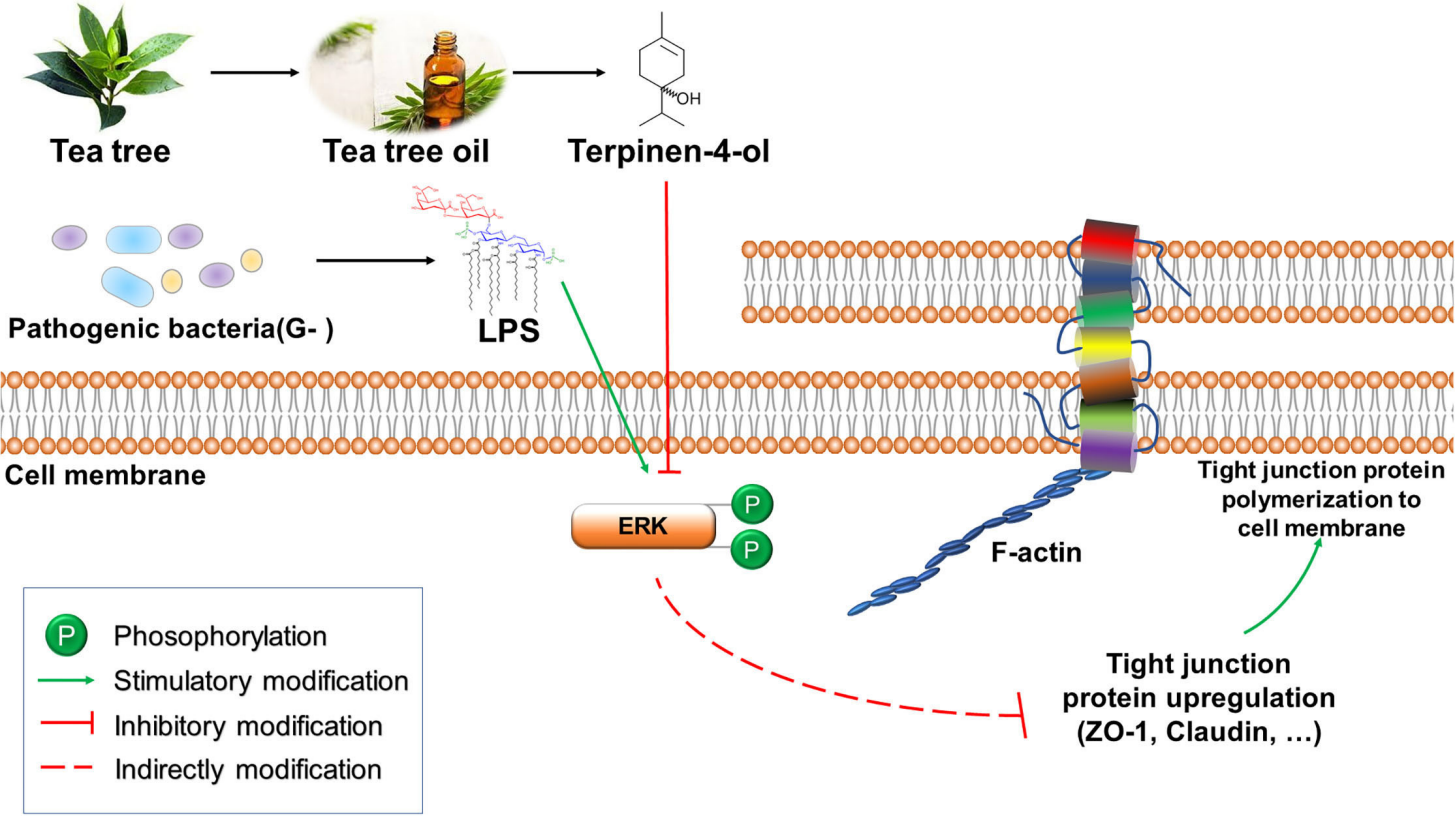
The putative signal pathway of terpinen-4-ol (TER) protects gut integrity in IPEC-J2 cells. TER attenuated LPS induces downregulation of tight junction proteins via the ERK1/2 signal pathway.

TER has attracted considerable interest as a bactericidal product in potential applications due to its unique biological activity, including antibacterial and antioxidant activities. Our study showed that TER protected IPEC-J2 cells against LPS-induced inflammation and DSS-induced colitis. Furthermore, we also showed the role of TJ proteins in LPS-or DSS-induced gut barrier damage via the ERK1/2-signaling pathway (Figure 10).

During growth and metabolic processes, cells secrete highly active biological oxidants, such as NO and hydroxyl radicals. Physiological doses of biological oxidants are essential for normal cellular metabolic processes and regulating various physiological functions. However, excessive oxidants will induce oxidative stress and subsequent cellular damage in the animal. A variety of pathogens and their secreted molecules, such as LPS, induce cells to produce high levels of oxidants (26). Essential oils containing TER can downregulate LPS-induced NO secretion (17), allowing normal cell growth and metabolism (27). In this study, we found a significant decrease in the viability of IPEC-J2 cells when exposed to LPS. On the contrary, the viability rate was significantly increased when pretreated with TER before LPS exposure. This phenomenon could be due to (i) pretreatment of TER significantly increased the IPEC-J2 cell proliferation rate, and (ii) TER protecting the intestinal barrier integrity, which attenuated the toxicity of LPS.

Inflammatory responses represent the initial pathological process during tissue damage in multiple diseases (28). Nuclear factor-kappa B (NF-κB) is activated when cells are activated by LPS, leading to an increase of intracellular inflammatory factors (29, 30). TER can significantly inhibit the activation of NF-κB, thereby reducing the inflammatory response (31). IL-6 and TNF-α were upregulated in IPEC-J2 cells after LPS stimulation, but pretreatment of the cells with TER, the above-mentioned phenomenon, disappeared. This indicates that TER exhibited significant anti-inflammatory activity.

When a pathogen invades the intestinal mucosal barrier, it can cause epithelial cell damage, activate inflammatory factors and mucosal immune activity, impair intestinal TJs (32), all of which can result in intestinal inflammation (33). This study demonstrated that TER improved intestinal barrier integrity. The mechanism may be related to the expression of TJ proteins, as TER pretreatment significantly ameliorated LPS- or DSS-induced damage to the epithelial integrity and decrease of tight junction proteins. The activating of p38 MAPK- and ERK1/2-pathways is related to inflammatory cytokine secretion in intestinal cells (34). The amelioration of *Lactobacillus pentosus*-induced colitis was based on inhibiting MAPKs and TJ protein expression (20, 35). In human corneal epithelial cells, TJ disruption occurred when the ERK1/2 pathway activated. In this study, TER attenuated LPS induced tight junction proteins decrease and ERK1/2 activation.

Similarly, pretreatment of IPEC-J2 cells with ERK1/2 inhibitor effectively inhibited the LPS-induced ERK1/2 activation and the TJ protein decrease. Moreover, TER attenuated colitis in DSS-treated mice was accompanied by TJ protein upregulation and inhibition of ERK1/2 phosphorylation in mice exposed to moderate and high TER doses, suggesting that ERK1/2 signaling is involved in TJ protein regulation by TER. In the LPS-induced inflammatory response of RAW264.7 macrophages, pedunculoside remarkably inhibited the phosphorylation of ERK1/2 to reduce the inflammatory cytokine production (32). However, as the phosphorylated ERK1/2 was significantly decreased in DSS alone and low dose TER treated mice, we speculate that cyclic changes in ERK1/2 expression may occur during the treatment process in the mice. For example, heat stress (HS) increased intestinal permeability and associated cyclic changes in TJ gene expression within 7 days (36), and ERK1/2 expression is quickly upregulated in acute HS but decreased sharply in pigs subjected to chronic HS (37).

In our study, TER remarkably improved DSS-induced colitis in mice and enhanced immune function. In addition, TER inhibited potential oxidative stress factors caused by DSS, thereby potentially protecting the intestinal barrier integrity by inhibiting intestinal cell apoptosis. Goblet cells are important mucus-secreting cells in the body (38). When goblet cells are reduced, mucus is secreted in insufficient quantities. The mucosal barrier function is impaired, resulting in inflammation (39). Our research also showed that TER ameliorated the DSS-induced decrease in the number of colonic goblet cells and the mucus layer thickness in mice. However, further study is required to elucidate the mechanism(s) by which goblet cells respond to TER.

In conclusion, the data demonstrate that TER attenuates LPS- or DSS-induced downregulation of TJ proteins via the ERK1/2-signaling pathway, which could be used as a novel therapeutic approach for treating IBD.

## Data Availability Statement

The original contributions presented in the study are included in the article/Supplementary Material, further inquiries can be directed to the corresponding author.

## Ethics Statement

The animal study was reviewed and approved by Animal Ethics Committee of Guangdong Ocean University.

## Author Contributions

XJ, YY, and BF designed the study. YY, BF, YH, JL, TY, LW, CH, XL, ZY, and XM participated in the experiments. YY, BF, XJ, SL, RG, and AA wrote and revised the manuscript. All authors approved the final manuscript.

## Funding

This study was supported by National Natural Science Foundation of China (Nos. 31472243 and 31902314), Natural Science Foundation of Guangdong Province, China (No. 019A1515011142), and Project of Enhancing School with Innovation of Guangdong Ocean University (No. GDOU230419057).

## Conflict of Interest

The authors declare that the research was conducted in the absence of any commercial or financial relationships that could be construed as a potential conflict of interest.

## Publisher's Note

All claims expressed in this article are solely those of the authors and do not necessarily represent those of their affiliated organizations, or those of the publisher, the editors and the reviewers. Any product that may be evaluated in this article, or claim that may be made by its manufacturer, is not guaranteed or endorsed by the publisher.
