# Supplementary material for: Tea Tree Oil Terpinen-4-ol Protects Gut Barrier Integrity by Upregulation of Tight Junction Proteins via the ERK1/2-Signaling Pathway
Source: Front Nutr. 2022 Jan 27;8:805612. doi: 10.3389/fnut.2021.805612 (PMC8829435; doi:10.3389/fnut.2021.805612)
Supplement: Supplementary file 1 [file Data_Sheet_1.docx]

Figure 5.

A：


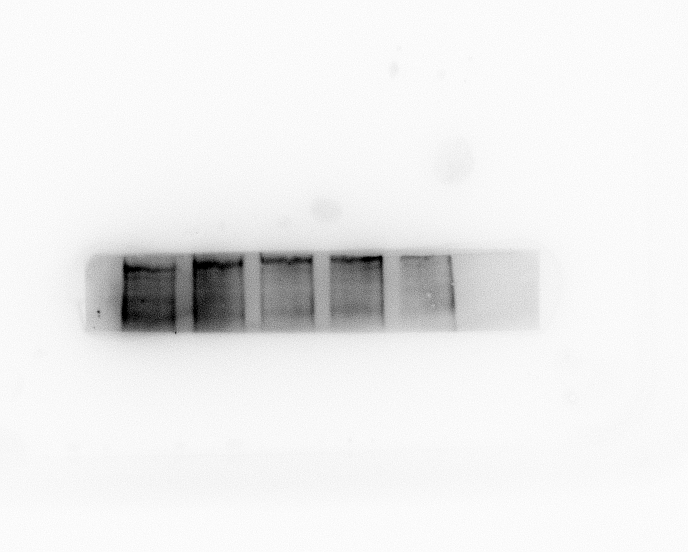
zo-1


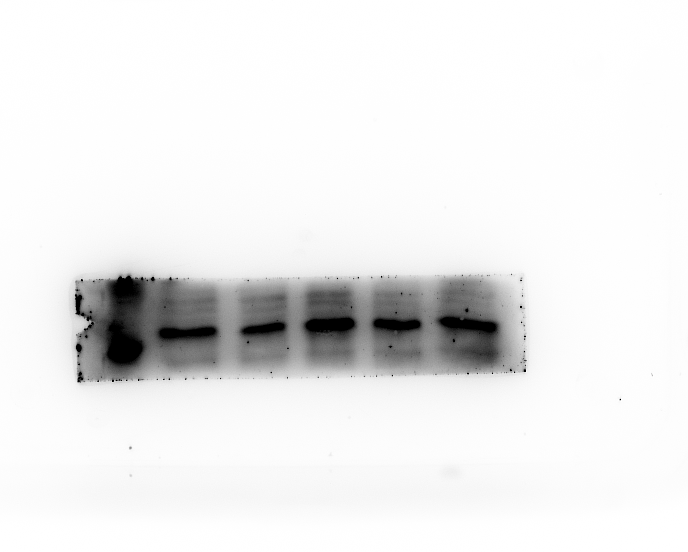
occludin

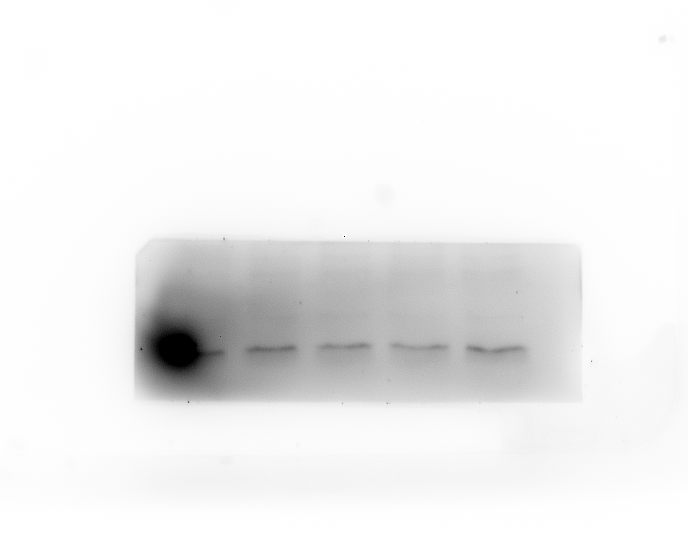
claudin-1


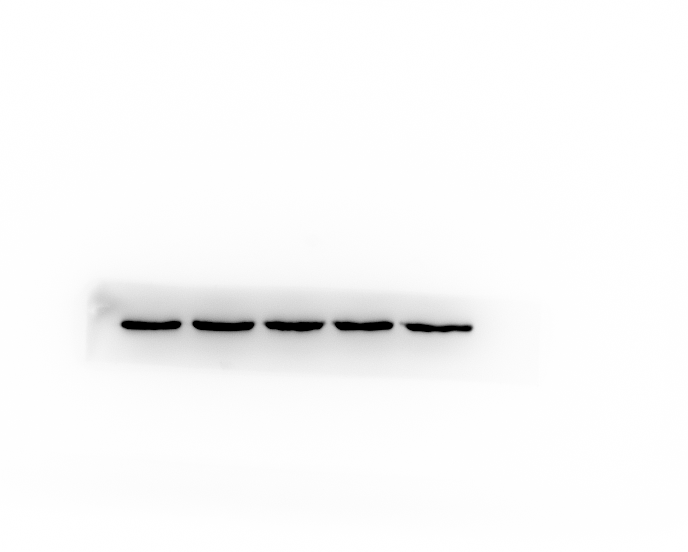
β-actin

B：



zo-1


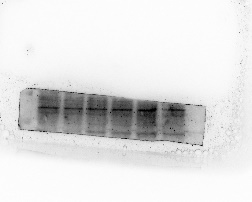
occulin



claudin-1


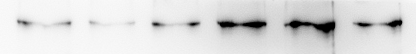
claudin-4



β-actin

C：



erk



P-erk



β-actin

D:



ERK



P-ERK



β-actin

Figure 6:

A：



ERK



P-ERK



β-actin

B：


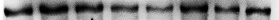
ZO-1


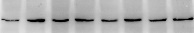
Occludin


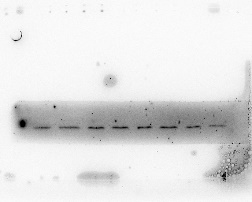
claudin-1



claudin-4



β-actin

Figure 9:

A：


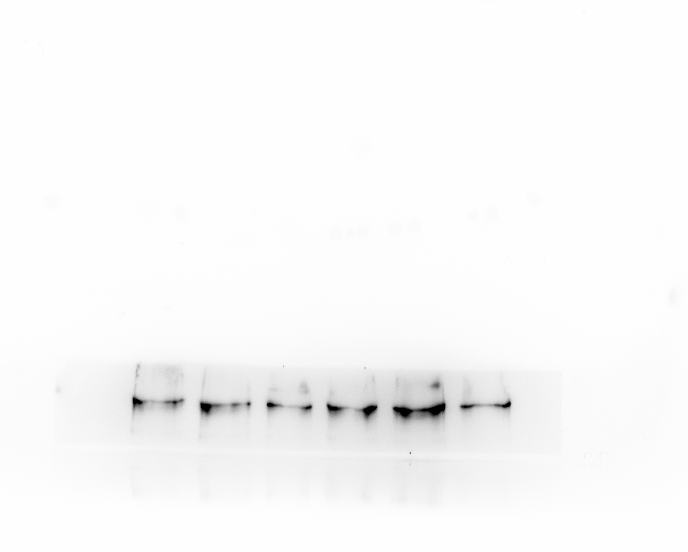
ZO-1



occludin


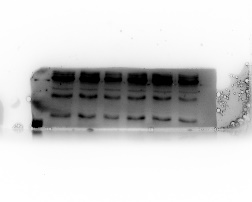
claudin-4



β-actin

B：



ERK


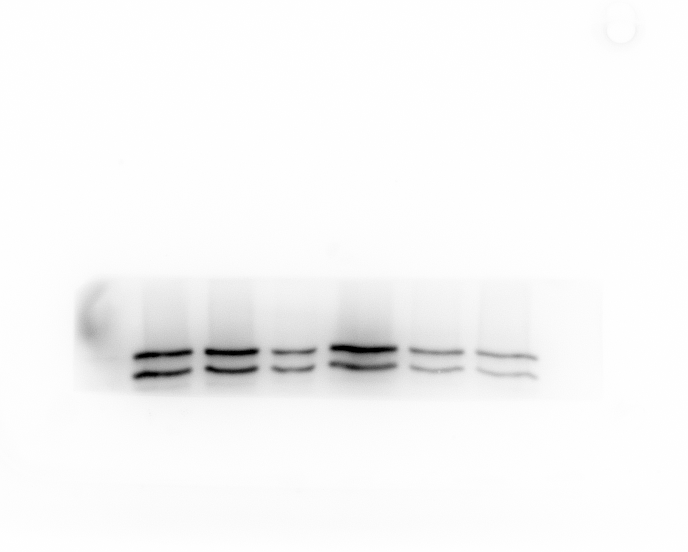
P-ERK


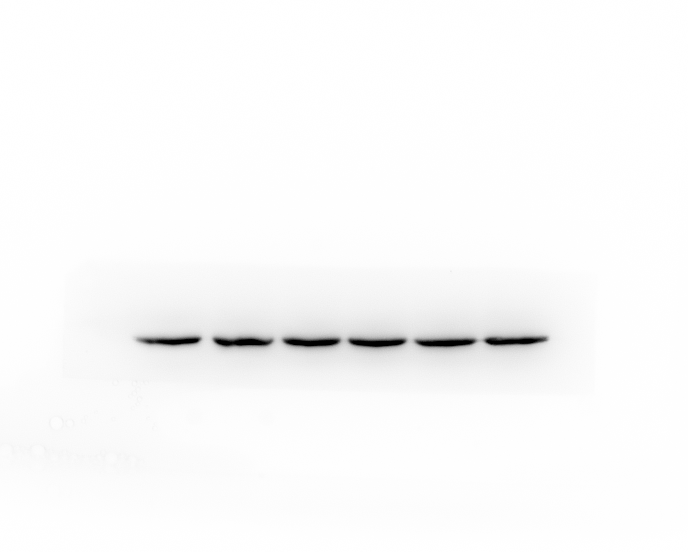


β-actin
